# Supplementary material for: SFC active reconfiguration based on user mobility and resource demand prediction in dynamic IoT-MEC networks
Source: PLoS One. 2024 Aug 1;19(8):e0306777. doi: 10.1371/journal.pone.0306777 (PMC11293735; doi:10.1371/journal.pone.0306777)
Supplement: Attachment — Submitted filename: 1259.docx [file pone.0306777.s001.docx]

Subject: Feedback on Article Evaluation

I hope this message finds you well. I have recently evaluated the article submitted to your esteemed magazine and would like to provide constructive feedback for your consideration.

The article delves into a significant debate crucial for the advancement of new-generation networks, a topic of considerable interest in current research. However, upon review, it is evident that the manuscript suffers from several critical issues that warrant attention.

1. **Abstract**:

- The abstract lacks a quantitative description to complement the qualitative evaluation presented.

- Algorithms compared with the proposed method should be explicitly mentioned.

- Address grammatical issues, and repetitions, and ensure clarity for enhanced coherence.

2. **Introduction**:

- Rectify grammatical errors and typos throughout the text.

- Improve clarity and coherence by enhancing organization and phrasing.

- Correct punctuation errors and unnecessary capitalizations.

- Condense repeated points for smoother flow.

- Define acronyms to aid readers' comprehension.

- Enhance engagement through more captivating language.

3. **Related Works**:

- Deepen the discussion of related works to address existing weaknesses.

- Categorize information for better structure and consider incorporating a table for advantages and disadvantages of works.

- Correct language errors and ensure consistency in terminology.

4. **System Model**:

- While the problem is well-formulated, analyze how the method overcomes identified limitations.

- Include an analysis of the algorithm's time complexity for a comprehensive understanding.

5. **Analysis of Simulation Results**:

- Address the following questions regarding the considered environment, user requirements, and method limitations.

- Evaluate the speed of convergence of machine learning algorithms and error functions.

- Update the dataset to reflect recent network service requirements.

- Consider the dynamic nature of the environment for more effective offline programming methods.

I believe addressing these concerns will significantly enhance the quality and impact of the article.

Warm regards,

Mohsen khani
